# The Effects of Dimethylsulfoxide and Oxygen on DNA Damage Induction and Repair Outcomes for Cells Irradiated by 62 MeV Proton and 3.31 MeV Helium Ions

**DOI:** 10.3390/jpm11040286

**Published:** 2021-04-08

**Authors:** Chun-Chieh Chan, Ya-Yun Hsiao

**Affiliations:** 1 Department of Electrical Engineering, National Chung Hsing University, Taichung 40227, Taiwan; andyccc0915@gmail.com; 2 Department of Radiology, Chung Shan Medical University Hospital, Taichung 40201, Taiwan; 3 Department of Medical Imaging and Radiological Sciences, Chung Shan Medical University, Taichung 40201, Taiwan

**Keywords:** reactive oxygen species, dimethylsulfoxide, DNA damage induction, hypoxia

## Abstract

Reactive oxygen species (ROS) play an essential role in radiation-induced indirect actions. In terms of DNA damage, double strand breaks (DSBs) have the greatest effects on the repair of DNA damage, cell survival and transformation. This study evaluated the biological effects of the presence of ROS and oxygen on DSB induction and mutation frequency. The relative biological effectiveness (RBE) and oxygen enhancement ratio (OER) of 62 MeV therapeutic proton beams and 3.31 MeV helium ions were calculated using Monte Carlo damage simulation (MCDS) software. Monte Carlo excision repair (MCER) simulations were used to calculate the repair outcomes (mutation frequency). The RBE values of proton beams decreased to 0.75 in the presence of 0.4 M dimethylsulfoxide (DMSO) and then increases to 0.9 in the presence of 2 M DMSO while the RBE values of 3.31 MeV helium ions increased from 2.9 to 5.7 (0–2 M). The mutation frequency of proton beams also decreased from 0.008–0.065 to 0.004–0.034 per cell per Gy by the addition of 2 M DMSO, indicating that ROS affects both DSB induction and repair outcomes. These results show that the combined use of DMSO in normal tissues and an increased dose in tumor regions increases treatment efficiency.

## 1. Introduction

Proton therapy and helium ion therapy have been used in radiation therapy (RT) and have attracted a lot interest owing to their abilities to deliver conformal dose into the tumor area and spare surrounding tissues [1,2]. This advantage is associated with the “Bragg curve”, by which the absorbed dose increases very gradually and then suddenly rise to a peak at the end of the track [3].

In RT, the mechanism for the induction of DNA damage for cells irradiated from ionizing radiations involving direct and indirect actions [4]. The atoms of DNA can be directly ionized or excited by the ionizing radiations, or the radiations can interact with water or cell medium to produce reactive oxygen species (ROS) to induce DNA damage [5]. For low linear energy transfer (LET) radiations, such as photons and protons, about two-thirds of DNA damage is induced via indirect actions, mostly by hydroxyl radicals (OH·) [4]. ROS induce DNA damage, DNA mutation, chromosome aberration and eventually cell transformation [5,6]. ROS such as hydroxyl radicals and superoxide (O_2_^•−^) can cause clustered DNA damage [7,8,9,10], which can be defined as DNA lesions generated by a single track of ionizing radiation [11]. Types of clustered lesions include two or more bases damage (BD), single-strand breaks (SSBs) and double strand breaks (DSBs) [12]. Among all types of DNA damage, DSB is probably a critical form of DNA damage and serves as the principle determinant of cell death [4,13].

For low-LET radiations, more than 90% of induced DNA damage is non-DSB clustered damage [14]. The repair process of non-DSB clustered lesions involves base excision repair (BER) pathways [15,16] and nucleotide excision repair (NER) [17,18] pathways. Monte Carlo simulations and experiments demonstrate that the point mutation frequency for BER pathways is proportional to radiation dose and LET [19], which implies that ROS also plays an important role in the repair process [20].

In RT, the use of dimethylsulfoxide (DMSO) as a ROS scavenger reduces the yields of DSB induction for both low and high-LET radiations [21] and increases cell survival [22,23]. Monte Carlo simulations and experiments also demonstrated that DMSO reduces the mutation frequency and increases the probability of correct repair [24,25]. Besides, DMSO can be used in RT to reduce the side effects [26].

Furthermore, most DNA damage formation involves the presence of O_2_ [27]. If O_2_ is present when DNA damage occurs, the damage is “ fixed ” and irreversible, which is termed oxygen fixation hypothesis [4]. O_2_ does not fix the damage directly; it modifies the pathway and the final chemical products [28]. That is, oxygen concentration has a special role in radiation oncology and radiosensitivity [29,30]. Hypoxia is reported to affect the biology of tumors, including the induction of DNA damage, the repair process and over-expression of some pro-survival genes [29,31,32]. There are several strategies to address hypoxia, notably, the use of RT with radiosensitizers such as nitric oxide inducers [33] and the targeting of the microenvironment of tumors, including the inhibition of hypoxia-inducible factor-1 genes [34].

Our previous study proposed that a ROS scavenger can enhance the difference in DSB induction and repair between normal and cancer cells during RT [25]. The combined use of DMSO on normal cells and radiosensitizers in the area of cancer cells can have a synergistic effect and further increase DNA damage yields and cell killing [35,36,37]. To our knowledge, MCDS is the only Monte Carlo code to be able to simulate under hypoxia and in the presence of DMSO. We have not found any other simulation to discuss the DNA profiles under hypoxia and also in the presence of DMSO. We present here the data for DSB induction for various concentrations of oxygen and DMSO for cells irradiated by 62 MeV proton beams and 3.31 MeV helium ions. The relative biological effectiveness (RBE), oxygen enhancement ratio (OER) [4] and the mutation frequency were simulated as a function of the concentration of DMSO.

## 2. Materials and Methods

### 2.1. Monte Carlo Damage Simulation (MCDS)

The MCDS is a fast algorithm that provides the yields and spatial information of clustered damage for cells irradiated with photons, light ions or heavy ions at a specific oxygen concentration [38,39,40]. MCDS is not a track structure simulation code and its algorithm generates DNA damage data similar to processes that are using computationally-expensive but detailed event-by-event simulations. MCDS used the DNA segment length as an ad hoc parameter in the simulation instead using the DNA model for a particular cell type [38]. The DNA segment length is adjustable to simulate the spectrum of DNA damage. Although not designed for a particular cell type, the results derived from MCDS have been shown to be comparable to the experimental results of cell types including Chinese hamster ovary cells, human fibroblasts, Hela cells and human bladder carcinoma [40].

MCDS also estimates the yields of DNA damage induction in the presence of DMSO and adjusts these according to the fraction of non-scavengeable DNA damage (*FNSD*) and the concentration at half-level (*CHMX*) [39]. *FNSD* represents the fraction of strand breaks and base damage that are not scavengeable, and *CHMX* can be translated as the concentration of DMSO that reduces to half of the yields of DSB induction. These parameter *FNSD* and *CHMX* were derived from fitting the measured DSB data to the results obtained by MCDS for ^60^Co γ-rays and 3.31 MeV helium ions [39]. The measured DSB data for cells irradiated by 62 MeV beams under aerobic conditions (21%) and in the presence of 0.064 M DMSO [41,42] has been used to derive *FNSD* and *CHMX* for MCDS simulations for proton ions [25]. Cells were respectively irradiated by ^60^Co γ-rays and 3.31 MeV helium ions with a dose 20−80 Gy [21] and by 62 MeV proton beams with a dose of 1−4 Gy [41,43]. As mentioned in the previous studies, the yields of DNA damage in the presence of DMSO were calculated using the values 0.52 and 0.21 M for *FNSD* and *CHMX*, respectively, for ^60^Co γ-rays [39]; and 0.52 and 0.07 M, respectively, for 62 MeV proton ions [25]; and 0.75 and 0.14 M, respectively, for 3.31 MeV helium ions [39].

### 2.2. Monte Carlo Excision Repair (MCER) Simulation

The MCER simulation simulates the key steps of repair pathways, that is, short-patch BER (SP BER), long-patch BER (LP BER), SP BER/NER, and LP BER/NER [19,44]. The MCER outputs the probability of the repair outcome whereby DNA clustered damage is repaired with a mutation for cells irradiated with electrons, protons and helium ions. The repair outcomes for cells in the presence of DMSO were calculated with the same values of parameter *FNSD* and *CHMX* for ^60^Co γ-rays (using the spectra of secondary electrons, see the session below), proton ions and helium ions, respectively, as described in the above section regarding MCDS simulations.

### 2.3. Calculation of DSB Induction and Mutation Frequency

The yield of DSB induction *Y* and mutation frequency *P* of cells irradiated by ^60^Co γ-rays were respectively calculated with the dose-weighted formula Equations (1) and (2) [45]:

(1) $$Y=\frac{\int_{0}^{\infty }dEY(E)\Phi (E){\mathrm{LET}}_{\infty }(E)}{\int_{0}^{\infty }dE\Phi (E){\mathrm{LET}}_{\infty }\left(E\right)}$$

(2) $$P=\frac{\int_{0}^{\infty }dEp(E)\Phi (E){\mathrm{LET}}_{\infty }(E)}{\int_{0}^{\infty }dE\Phi (E){\mathrm{LET}}_{\infty }\left(E\right)}$$

where Φ(*E*) is the energy fluence of the secondary electrons of energy *E* that were taken from a previous study [45]. *Y*(*E*) represents the yield of the DSB induction per Gy per gigabase pairs (per Gy per Gbp) with the secondary electrons of energy *E*. *P* represents the frequency of repair with at least one base substitution averaged over all types of DNA damage (Semenenko et al., 2005). *P*(*E*) represents the mutation frequency (per Gy per Gbp) with secondary electrons of energy *E*. The sources of the values for unrestricted LET (stopping power) have been described previously [45].

### 2.4. OER and RBE

The OER is defined as the ratio of DSB yield under aerobic conditions to that under hypoxic conditions [46]. This study defines the OER as the ratio of DSB yield under 21% O_2_ to the yield under the hypoxic condition, 0.001% O_2_.

RBE is interpreted as the ratio of the DSB yields of cells irradiated with different radiation sources, as shown below:

(3) $$RBE=\frac{D_{\gamma }}{D_{R}}=\frac{\Sigma_{R}}{\Sigma_{\gamma }}$$

The subscripts, *R* and *γ*, denote radiation particles (i.e., proton ions and helium ions) and γ-rays, respectively. The DSB yield for ^60^Co γ-rays is the reference for all reported RBE values.

## 3. Results

Table 1 shows the absolute yields of DSB induction for cells irradiated with 62 MeV proton ions in the absence or presence of 0.1–1 M DMSO. The results of this study showed that the yields of all types of DNA damage induction decreased as the concentration of DMSO increased. Both BD and SSB decreased by 19–24% in the presence of 0.1 M DMSO, which was less than those of complex damage where DSB and DSB^++^ were reduced by 45–75%. When the concentration increased to 0.5 M or more, all types of DNA damage approximately reach to constant values and were not sensitive to changes in the concentration of DMSO.

Table 2 shows the yields of DSB induction in the presence of DMSO and under 0.1–21% O_2_ and provides a comparison of DNA damage profiles to mimic the effects of DMSO under hypoxia. In the presence of 0.1 M DMSO, BD and SSB decreased by 24–30% and DSB and DSB^++^ decreased by 53–81% under 2% O_2_. The yields of all types of DNA damage were slightly reduced (less 10%) when the oxygen concentration decreased from 21% to 2%. When the oxygen concentration decreased to 0.1%, BD and SSB decreased by 42–49% and DSB and DSB^++^ decreased by 76–97%, indicating that a larger reduction was observed in severely hypoxic conditions (~0.1% O_2_).

Furthermore, we compared the trends of DSB induction under 2% O_2_ (Table 3) and 0.1% (Table 4) in the presence of 0–1 M DMSO. In Table 3, under 2% O_2_, BD and SSB decreased by at most 38–44% (1 M) while complex damage decreased by 71–94% (1 M). Under severe hypoxic condition of 0.1% O_2_, BD and SSB decreased by 42–49% and complex damage decreased by 76–97% in the presence of 0.1 M DMSO (Table 4). The yields of simpler damage, such as BD and SSB, were reduced to 53–60% in the presence of 1 M DMSO while complex DSB damage, i.e., DSB^++^ only slightly increased to 98%. In terms of DSB induction, a low concentration of DMSO (~0.1 M) inhibits at least 45% of DSB induction (Table 1) and a higher concentration may improve slightly. In severe hypoxia (0.1% O_2_), DMSO has limited effects on DSB induction but exhibits stronger influence on simpler damage, such as BD and SSB (Table 4).

**Figure 1:**
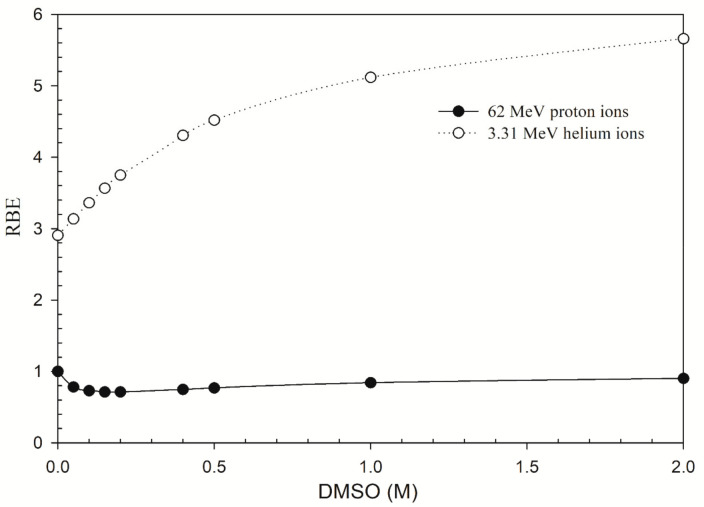
Relative biological effectiveness (RBE) as a function of the concentration of DMSO (0–2 M). Monte Carlo damage simulation (MCDS)-derived RBE values for DSB induction were shown for cells irradiated by 62 MeV proton ions (LET = 1.051 keV/μm) and 3.31 MeV helium ions (LET = 120 keV/μm).

**Figure 2:**
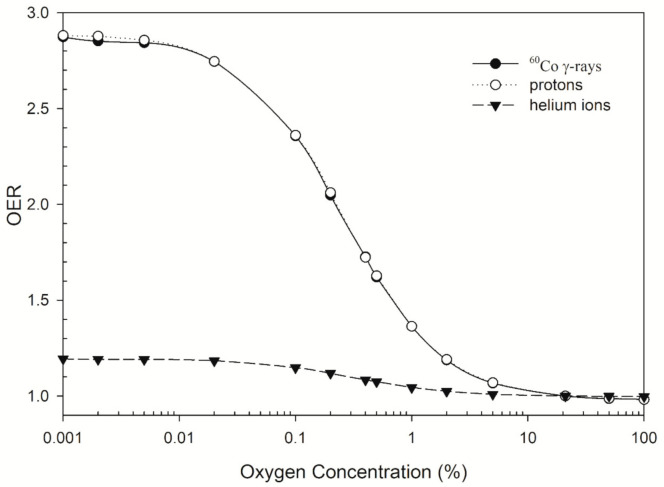
Oxygen enhancement ratio (OER) as a function of oxygen concentration (0.001–100% O_2_). MCDS-derived OER values were plotted for cells irradiated by ^60^Co γ-rays (LET = 2.4 keV/μm), 62 MeV proton ions (LET = 1.051 keV/μm) and 3.31 MeV helium ions (LET = 120 keV/μm).

In Figure 1, the RBE values for DSB induction for cells irradiated by 62 MeV proton ions decreased to 0.75 in the presence of 0.4 M DMSO and then increased to 0.9 in the presence of 2 M DMSO. On the contrary, the RBE values for DSB induction for cells irradiated by 3.31 MeV helium ions increased from 2.9 to 5.7 when the concentration of DMSO increased to 2 M, indicating a stronger contribution from direct action. The effects of oxygen on DSB induction are shown in Figure 2. For low-LET radiations, the OER values for DSB induction for cells irradiated by proton ions have similar profiles to those for ^60^Co γ-rays: both values decreased from 2.9 to 1.0 when the oxygen concentration increased from 0.001% to 21%. For high-LET radiations, the OER value for 3.31 MeV helium ions decreased from 1.2 to 1.0 over the same range (0.001–100% O_2_). These results showed that about only one-third of DSB yields under aerobic conditions (21% O_2_ or higher) were induced in the severe hypoxia conditions (~0.1% O_2_) for proton ions and ^60^Co γ-rays while 83% of DSB yields were induced for helium ions.

**Figure 3:**
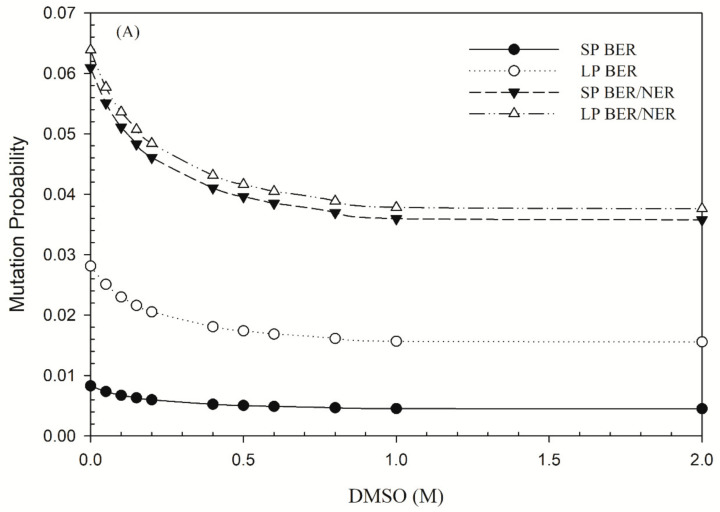

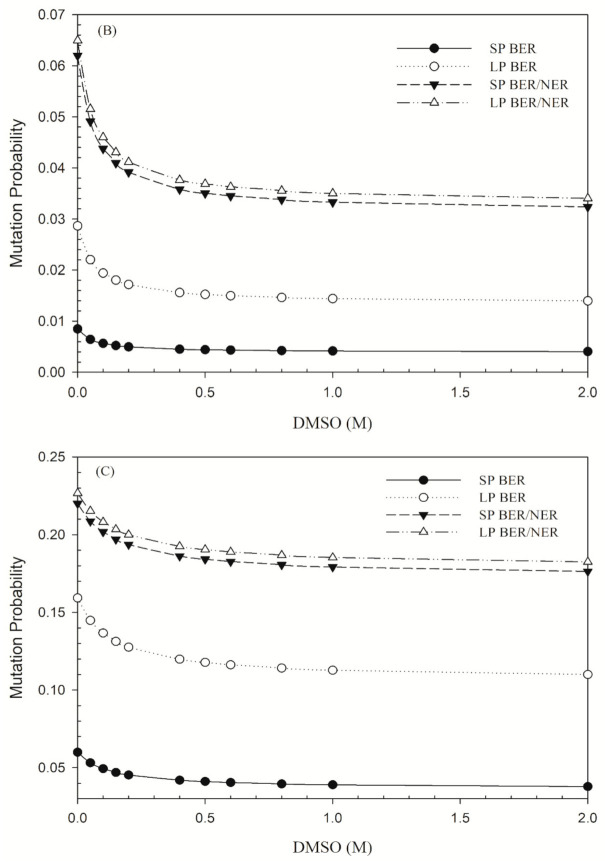
Mutation frequency (per Gy per cell) as a function of the concentration of DMSO for cells irradiated by (**A**) ^60^Co γ-rays (LET = 2.4 keV/μm), (**B**) 62 MeV proton ions (LET = 1.051 keV/μm) and (**C**) 3.31 MeV helium ions (LET = 120 keV/μm). All four pathways (SP BER, LP BER, NER/SP BER and NER/LP BER) were simulated.

In terms of repair outcomes, Figure 3 shows the mutation frequency as a function of the concentration of DMSO. For ^60^Co γ-rays or protons, DMSO produced a significant reduction in the mutation frequency (Figure 3A,B). In the presence of 0.5 M DMSO, the mutation frequency reduced by 35–39% and 43–48% for all four pathways for ^60^Co γ-rays and protons, respectively. For helium ions, the mutation frequency reduced by 16–31% (Figure 3C).

## 4. Discussion

This study evaluated the DSB induction and repair outcomes in the presence of DMSO and/or under hypoxia. The MCDS-derived results for DSB induction for low- and high-LET radiations have been compared with experimental data and track structure simulations elsewhere [38,39,40,45,47,48], including the data for the presence of DMSO or under hypoxia [24,25]. Recent experimental data showed that the yields for DSB induction in WiDr (human colon carcinoma) cells irradiated with 6 MV X-rays in the presence of 0.28 M DMSO decreased to 62% using pulse-field gel electrophoresis assay [22] while MCDS simulation showed that the DSB yields reduced to 54%. Other data reported that Chinese hamster ovary cells treated with 0.5% (0.064 M) DMSO reduced to 80–86% using the measurement of 53BP1 foci [49] while MCDS predicted that the DSB yields reduced to 79%, which was in good agreement with the experimental results. In terms of repair outcomes, the experimental data showed that the mutation frequency for 0.88 MeV protons was 0.023 per Gy per cell [19,50] while MCDS predicted that the mutation frequency was in the range of 0.025–0.143 per Gy per cell for all four pathways, which was very close to the measured value.

In Figure 1, the RBE values for proton ions are less than one when the concentration of DMSO increases, which shows that DMSO provides greater protection for protons than ^60^Co γ rays. However, the difference in LET may affect the estimation of RBE. The LET for the spectrum for cells irradiated by ^60^Co γ-rays is 2.4 keV/μm [25] and the LET of 62 MeV protons at entrance is 1.051 keV/μm [25]. Indirect effects may have a larger contribution to the yields of DNA damage induced by proton irradiations as these proton beams have a lower LET. Assuming that DMSO interferes with indirect action only, our data suggested the contribution of indirect action on DSB induction for proton and ^60^Co γ rays irradiation was about 66% (Table 1) and 55% (Figure 1), respectively, at 0.5 M DMSO, which agreed well with the experimental value for ^60^Co γ-rays, 50% [21]. It was reported that the contribution of indirect action on cell killing was about 62% for EMT6 mouse sarcoma cells irradiated by X-ray irradiation (LET = 2 keV/μm) and 53% for 225 MeV proton beam irradiation (LET = 1.92 keV/μm) (both at 0.5 M DMSO) [51], respectively. Other experimental data showed that the contribution of indirect action on cell killing for human leukemia HL-60 cells was about 85% for X-rays and 92% for carbon ions (LET = 20 keV/μm) irradiations [52]. The protective effects of DMSO may vary due to different biological endpoint and cell type and exhibit a LET-dependency only for high-LET heavy ions [52]. The protective effects of DMSO seem to be more compelling for cell survival than DSB induction. It has been showed that the RBE for DSB induction is generally smaller than that for cell survival [48]. The protective effects of DMSO at the cellular levels lead to a larger impact on cell survival.

Figure 2 shows that the OER values for ^60^Co γ-rays and protons for DSB induction are around 2.9 and are in good agreement with the experimental values for the OER values for DSB induction for X-rays, 2.9−3.4 [53,54]. In the presence of 2 M DMSO, Sapora et al. (1991) reported that the OER values for SSB and DSB induction for cells irradiated with X-rays were 2.0 and 2.2, respectively [53] while MCDS-derived values were 1.7 and 2.9, respectively. A combination of hypoxia and DMSO reduces the yields of all types of DNA damage but the predicted OER values for DSB induction using MCDS may be overestimated. Moreover, MCDS may not be used in some cell types. That is, the cell types used in the data to be the benchmark for MCDS are all anchorage-dependent, including Chinese hamster ovary cells, normal human fibroblast cells, Hela cells and human bladder carcinoma [40]. If the cell type is suspended and/or highly radiosensitive such as blood cells, then MCDS may not correctly predict the DNA profiles.

The protective effects of DMSO in reducing DNA damage induction have previously been ascribed to its role as a ROS scavenger [55]. However, studies also showed its toxicity [56] and diverse effects on cell differentiation [57], but mostly were related to the administered doses. The concentrations of DMSO used in the literatures were mostly within 2 M [21,23,53,58,59,60] but it was reported to cause gross molecular changes, reduce cell viability and induce cytotoxicity for the concentrations above 1% (0.128 M) [57,61]. The concentrations below 0.5% (0.064 M) were regarded as non-toxic [49] and only negligible toxicity was reported if the concentration increased to 1 M [58]. Other studies show that the cytotoxicity induced by DMSO probably depends on cell type [62,63]. Our results suggest the concentration of DMSO used for RT could be in the range of 0.1–0.5 M, which might be optimal for the balance in reducing DNA damage induction and maintaining low toxicity.

The mechanism of protection by DMSO was assumed to involve the generation of the secondary radicals (e.g., methylperoxyl radicals) [64], from the reaction of DMSO with ROS. The ROS such as OH· are very effective in DSB induction and generate DSBs [7,9]. The reaction of DMSO with ROS produces the secondary radicals (e.g., methylperoxyl radicals) [64]. These radicals reacted very fast with oxygen (if present) and yield unreactive peroxyl products which are less detrimental in DSB induction than the OH free radicals, leading a decrease in damage yields [65,66]. This suggests that the scavenging ability of DMSO is limited in hypoxia and the number of secondary free radicals other than OH·also increases [65]. Under hypoxic conditions and in the presence of DMSO, these secondary free radicals or DNA radicals reacted with endogenous thiols, such as glutathione, which has been described as chemical repair [27]. Our data indicated that the yields of all types of DNA damage in anoxic cells reduce slightly (~12−21%) in the presence of 0.1 M DMSO (Table 4) in contrast to the larger reduction (~19−62%) in oxygenated cells, indicating that DMSO has a limited scavenging effect in hypoxia.

Recently, several studies also indicate that DMSO acts to facilitate the repair of DNA damage [26,49,59,67]. Radioprotection due to the presence of DMSO is manly from the facilitation of DNA damage repair rather than through the suppression of indirect actions [26,49]. Bajinskis et al. (2013) reported that DMSO affects primarily on all DNA repair pathways [59]. Yang et al. suggested that DMSO may facilitate the DSB repair pathways only [26]. Our study showed that the mutation probabilities of BER and NER pathways for ^60^Co γ-rays, proton ions and high-LET helium ions were all respectively reduced by 35–39%, 43–48% and 16–31% in the presence of 2 M DMSO, indicating that DMSO might also alleviate the BER and NER repair pathways.

DMSO and radiosensitizers both have been used in RT for decades [55,68]. DMSO has been used to reduce the side effects in RT, in in vitro [41,52,53,58] and in vivo studies [26,69]. We have not found any clinical trial that uses DMSO during RT. However, DMSO has been used in clinical trials in dermatology [70] and pain reliever [71,72]. Our results suggest that DMSO can be applied after the irradiation at different timings to reduce the yields of DSB induction and improve the outcomes of DNA damage repair in the normal tissue area while radiosensitizers can be used in the tumor regions to increase the concentration of oxygen. We can also add oxygen mimetics [37] as a substituent for oxygen in the process of “fixing” DNA damage. As shown in Table 3 and Table 4, the yields of DSB induction reduce by 16% when the oxygen concentration decreases from 21% to 2%. However, if DMSO is used, even in a low concentration such as 0.1 M, the portion of the yield of DSB induction (reduced by DMSO) increases from 16% to 56%. Therefore, the combined use of DMSO in normal cells along with radiosensitizers increases the difference in the yields of DSB induction. The protective effects of DMSO can reduce complications for surrounding normal cells induced by a single dose in the tumor area [73] or fractioned doses applied on the head [26].

Alternatively, a larger irradiated volume may be applied in RT along with the use of higher concentrations of DMSO in the outer regions. That is because that the degree of protection of DMSO is associated with the concentrations of DMSO and oxygen. The oxygen can be seen as a biomarker to distinguish normal and tumor cells: the median oxygen level in most normal tissues is around 4–7.5% while the level in tumors is 0.3–4.2% [74]. For example, the yield of DSB induction for normal cells (assuming 5% O_2_) irradiated by proton irradiations in the presence of 0.5 M DMSO is 2.68 per Gy per Gbp while that for tumors cells (assuming 2% O_2_) in the presence of 0.1 M DMSO is 3.66 per Gy per Gbp (Table 3). Using concentration gradient of DMSO, the yields of DSB induction for tumors could be higher than those for normal cells, therefore it is possible to improve the treatment efficiency and protect healthy cells simultaneously. In practice, it has been reported that mice were injected intraperitoneally with DMSO (4 g/Kg) 1 h before head-only irradiations [26]. DMSO can also be injected subcutaneously into the outer region of the irradiated volume [75] and the injected DMSO may be diffused slowly to form the concentration gradient.

In summary, this study evaluated the effects of indirect action by ROS and oxygen. The DNA damage profiles were derived for cells irradiated by low-LET proton beams in the presence of various concentrations of DMSO and oxygen. The RBE and OER of proton beams and helium ions were calculated as a function of the concentration of DMSO. These results show that DMSO provides a significant protection, even at a low concentration such as 0.1 M, but has limited scavenging effects in hypoxic conditions. Using oxygen as a biomarker, the concentration gradient of DMSO can be used to protect healthy cells to allow a larger irradiated volume during the treatment.

## Figures and Tables

**Table 1:** Absolute Yields of Double Strand Break (DSB) Inductions for Cells Irradiated with Proton Ions (Linear Energy Transfer (LET) = 1.051 keV/μm) in the Presence of Various Concentrations of DMSO.

Absolute Yields (per Gy per Gbp)	BD ^a^	SSB ^a^	SSB^+ a^	2SSB ^a^	DSB ^a^	DSB^+ a^	DSB^++ a^	Total SSB ^a^	Total DSB ^a^	Total Damage
proton ^b^	421.03 ± 0.04	177.77 ± 0.02	8.04 ± 0.00	1.01 ± 0.00	7.19 ± 0.01	0.99 ± 0.00	0.12 ± 0.00	186.83 ± 0.02	8.29 ± 0.01	616.15 ± 0.05
proton + 0.1 M DMSO	341.22 ± 0.04	135.00 ± 0.02	4.20 ± 0.00	0.39 ± 0.00	3.95 ± 0.01	0.38 ± 0.00	0.03 ± 0.00	139.59 ± 0.02	4.36 ± 0.01	485.17 ± 0.04
	(19%↓)	(24%↓)	(48%↓)	(62%↓)	(45%↓)	(61%↓)	(75%↓)	(25%↓)	(47%↓)	(21%↓)
proton + 0.5 M DMSO	292.05 ± 0.03	111.85 ± 0.02	2.77 ± 0.00	0.20 ± 0.00	2.64 ± 0.01	0.20 ± 0.00	0.01 ± 0.00	114.83 ± 0.02	2.86 ± 0.01	409.74 ± 0.03
	(31%↓)	(37%↓)	(65%↓)	(80%↓)	(63%↓)	(79%↓)	(90%↓)	(39%↓)	(66%↓)	(33%↓)
proton + 1 M DMSO ^b^	281.33 ± 0.03	107.09 ± 0.01	2.54 ± 0.00	0.18 ± 0.00	2.45 ± 0.01	0.17 ± 0.00	0.00 ± 0.00	109.80 ± 0.01	2.62 ± 0.01	393.75 ± 0.03
	(33%↓)	(40%↓)	(68%↓)	(82%↓)	(66%↓)	(82%↓)	(100%↓)	(41%↓)	(68%↓)	(36%↓)

^a^ As mentioned above, DNA damage includes base damages (BD), single-strand breaks (SSBs), DSBs, two or more strand breaks on the same strand (SSB^+^), two or more strand breaks on opposite strands that dot not constitute a DSB (2SSB), DSBs with additional break(s) on a strand within 10 base pairs (DSB^+^) and more than one DSB within 10 base pairs (DSB^++^). The total SSB refers to a combination of SSB, SSB^+^ and 2SSB; and the total DSB refers to a combination of DSB, DSB^+^ and DSB^++^. The DNA damage yields were converted in to the unit per Gy per Gbp using the factor 6 Gbp per cell for a typical mammalian cell [38]. ^b^ The data were derived from the study by Luo et al. (2020) [25].

**Table 2:** Absolute Yields of DSB Inductions for Cells Irradiated with Proton Ions (LET = 1.051 keV/μm) under Various Concentrations of Oxygen (O_2_) and at 0.1 M DMSO.

Absolute Yields (per Gy per Gbp)	BD	SSB	SSB^+^	2SSB	DSB	DSB^+^	DSB^++^	Total SSB	Total DSB	Total Damage
proton ^a^	421.03 ± 0.04	177.77 ± 0.02	8.04 ± 0.00	1.01 ± 0.00	7.19 ± 0.01	0.99 ± 0.00	0.12 ± 0.00	186.83 ± 0.02	8.29 ± 0.01	616.15 ± 0.05
proton + 0.1 M DMSO + 21% O_2_	341.22 ± 0.04	135.00 ± 0.02	4.20 ± 0.00	0.39 ± 0.00	3.95 ± 0.01	0.38 ± 0.00	0.03 ± 0.00	139.59 ± 0.02	4.36 ± 0.01	485.17 ± 0.04
	(19%↓)	(24%↓)	(48%↓)	(62%↓)	(45%↓)	(61%↓)	(75%↓)	(25%↓)	(47%↓)	(21%↓)
proton + 0.1 M DMSO + 2% O_2_	320.19 ± 0.03	124.91 ± 0.02	3.53 ± 0.00	0.30 ± 0.00	3.34 ± 0.01	0.29 ± 0.00	0.02 ± 0.00	128.73 ± 0.02	3.66 ± 0.01	452.58 ± 0.04
	(24%↓)	(30%↓)	(56%↓)	(70%↓)	(53%↓)	(70%↓)	(81%↓)	(31%↓)	(56%↓)	(27%↓)
proton + 0.1 M DMSO + 0.1% O_2_	244.96 ± 0.03	91.24 ± 0.01	1.76 ± 0.00	0.11 ± 0.00	1.74 ± 0.01	0.10 ± 0.00	0.00 ± 0.00	93.12 ± 0.01	1.85 ± 0.01	339.92 ± 0.03
	(42%↓)	(49%↓)	(78%↓)	(89%↓)	(76%↓)	(90%↓)	(97%↓)	(50%↓)	(78%↓)	(45%↓)

^a^ The data were derived from the study by Luo et al. (2020) [25].

**Table 3:** Absolute Yields of DSB Inductions for Cells Irradiated with Proton Ions (LET = 1.051 keV/μm) under 2% Oxygen (O_2_) and at Various Concentrations of DMSO.

Absolute Yields (per Gy per Gbp)	BD	SSB	SSB^+^	2SSB	DSB	DSB^+^	DSB^++^	Total SSB	Total DSB	Total Damage
proton ^a^	421.03 ± 0.04	177.77 ± 0.02	8.04 ± 0.00	1.01 ± 0.00	7.19 ± 0.01	0.99 ± 0.00	0.12 ± 0.00	186.83 ± 0.02	8.29 ± 0.01	616.15 ± 0.05
proton + 2% ^a^	399.55 ± 0.04	165.47 ± 0.02	6.78 ± 0.00	0.78 ± 0.00	6.14 ± 0.01	0.76 ± 0.00	0.08 ± 0.00	173.03 ± 0.02	6.99 ± 0.01	579.57 ± 0.04
	(5%↓)	(7%↓)	(16%↓)	(23%↓)	(15%↓)	(23%↓)	(31%↓)	(7%↓)	(16%↓)	(6%↓)
proton + 0.1 M DMSO +2%	320.19 ± 0.03	124.91 ± 0.02	3.53 ± 0.00	0.30 ± 0.00	3.34 ± 0.01	0.29 ± 0.00	0.02 ± 0.00	128.73 ± 0.02	3.66 ± 0.01	452.58 ± 0.04
	(24%↓)	(30%↓)	(56%↓)	(70%↓)	(53%↓)	(70%↓)	(81%↓)	(31%↓)	(56%↓)	(27%↓)
proton + 1 M DMSO +2%	262.45	98.73	2.12	0.14	2.06	0.13	0.01	100.99	2.20	365.65
	(38%↓)	(44%↓)	(74%↓)	(87%↓)	(71%↓)	(87%↓)	(94%↓)	(46%↓)	(73%↓)	(41%↓)

^a^ The data were derived from the study by Luo et al. (2020) [25].

**Table 4:** Absolute Yields of DSB Inductions for Cells Irradiated with Proton Ions (LET = 1.051 keV/μm) under 0.1% Oxygen (O_2_) and at Various Concentrations of DMSO.

Absolute Yields (per Gy per Gbp)	BD	SSB	SSB^+^	2SSB	DSB	DSB^+^	DSB^++^	Total SSB	Total DSB	Total Damage
proton ^a^	421.03 ± 0.04	177.77 ± 0.02	8.04 ± 0.00	1.01 ± 0.00	7.19 ± 0.01	0.99 ± 0.00	0.12 ± 0.00	186.83 ± 0.02	8.29 ± 0.01	616.15 ± 0.05
proton + 0.1%	316.26 ± 0.03	123.07 ± 0.02	3.42 ± 0.00	0.28 ± 0.00	3.25 ± 0.01	0.28 ± 0.00	0.02 ± 0.00	126.76 ± 0.02	3.55 ± 0.01	446.58 ± 0.04
	(25%↓)	(31%↓)	(57%↓)	(73%↓)	(55%↓)	(72%↓)	(85%↓)	(32%↓)	(57%↓)	(28%↓)
proton + 0.1 M DMSO +0.1%	244.96 ± 0.03	91.24 ± 0.01	1.76 ± 0.00	0.11 ± 0.00	1.74 ± 0.01	0.10 ± 0.00	0.00 ± 0.00	93.12 ± 0.01	1.85 ± 0.01	339.92 ± 0.03
	(42%↓)	(49%↓)	(78%↓)	(89%↓)	(76%↓)	(90%↓)	(97%↓)	(50%↓)	(78%↓)	(45%↓)
proton + 1 M DMSO +0.1%	196.90 ± 0.02	71.52 ± 0.01	1.06 ± 0.00	0.05 ± 0.00	1.05 ± 0.00	0.05 ± 0.00	0.00 ± 0.00	72.63 ± 0.01	1.10 ± 0.00	270.63 ± 0.02
	(53%↓)	(60%↓)	(87%↓)	(95%↓)	(85%↓)	(95%↓)	(98%↓)	(61%↓)	(87%↓)	(56%↓)

^a^ The data were derived from the study by Luo et al. (2020) [25].
